# Effect of Thrombin-Induced MCP-1 and MMP-3 Production Via PAR1 Expression in Murine Intervertebral Discs

**DOI:** 10.1038/s41598-018-29669-z

**Published:** 2018-07-27

**Authors:** Yoshihiro Takayama, Takashi Ando, Jiro Ichikawa, Hirotaka Haro

**Affiliations:** Department of Orthopaedic Surgery, Faculty of Medicine, University of Yamanashi, 1110 Shimokato, Chuo, Yamanashi, 409-3898 Japan

## Abstract

Structural changes in nucleus pulposus cells induce intervertebral disc (IVD) degeneration as a consequence of cytokine generation, biochemical products, and changes in the local environment. We have previously shown that inflammatory cytokines induce murine IVD (mIVD) angiogenesis and macrophage migration. Although the physiological roles of thrombin, a known proinflammatory factor, are documented, its relationship to IVD degeneration remains largely unexplored. Thrombin mediates cellular responses via the activation of protease-activated receptors such as PAR1 which has been studied in numerous cell types, but not extensively in IVD cells. This study was designed to investigate the endogenous expression of thrombin, tissue factor, and PAR1 in cultured coccygeal mIVDs. Thrombin exclusively induced MCP-1 via the MAPK-ERK and PI3K-AKT pathways. MCP-1 produced by mIVDs induced macrophage migration and thrombin treatment increased MMP-3 production to induce mIVD degeneration. These effects of thrombin on mIVDs were abrogated by a PAR1 inhibitor and suggest that thrombin may be a novel factor capable of stimulating cytokine activity implicated in the regulation several aspects of mIVDs. Mechanisms governing mIVDs, which are regulated by thrombin/PAR1 signaling, require elucidation if our understanding of IVD degenerative mechanisms is to advance.

## Introduction

Degeneration of intervertebral discs (IVDs) is one cause of low back pain and various reports about the mechanism of action exist^1–3^. Risk factors for IVD degeneration include—but are not limited to—older age, manual labor, and smoking. Intervertebral discs represent the largest avascular organ in the human body. Disc nourishment is predominantly via diffusion from blood vessels extending from the upper and lower vertebral bodies to the subchondral plate and to the cartilage of the endplates (CEPs). Additional nourishment can be delivered via blood flowing from vessels surrounding the IVD to the outer periphery of the annulus fibrosus (AF). Intervertebral disc degeneration is a consequence of structural changes in the nucleus pulposus (NP) cells caused by cytokine generation, biochemical products, and changes in the local environment^4,5^. In the early phase of degeneration, environmental changes in the vicinity of NP cells, such as CEP ossification, can divert the blood flowing around vertebral bodies, decreasing the supply of nutrients and oxygen to cause cell death and convert the NP cell population to chondrocyte-like cells which migrate from inner AF or CEP cells^6–8^. In the late phase of degeneration, angiogenesis can be observed, accompanied by nerve infiltration, a normoxic environment around NP cells, and a resultant decrease in the activity of NP cells^9^. With an increase in age, tumor necrosis factor (TNF)-α, IL (interleukin)-1α, and IL-1β naturally increase which can promote the synthesis of matrix metalloproteinases (MMPs) and subsequent IVD degeneration. We have previously reported that herniated disc tissue contains abundant macrophage infiltration and high levels of MMPs, particularly MMP-3 and MMP-7. Chondrocytic MMP-3, but not MMP-7, was required for disc resorption based on assays that showed a reduction in wet weight and proteoglycan content after 72 h of co-culture with macrophages^10^. Furthermore, disc tissue from 64-week-old MMP-3-deficient mice did not exhibit IVD matrix degradation^11^. These results suggest that MMP-3 plays a pivotal role in IVD degradation. In other previous studies, mechanical stress has also been shown to cause IVDs to release various inflammatory cytokines such as IL-6, C-X-C motif chemokine ligand 8 (CXCL-8), IL-15, monocyte chemoattractant proteins-1 and -3 (MCP-1 and MCP-3, respectively), TNF-α, and nerve growth factor^2,3,6,12^.

We have previously shown that angiogenesis and macrophage cell migration play an important role in the degeneration of herniated discs by stimulating the inflammatory cytokines MCP-1, TNF-α, TNF-like weak inducer of apoptosis (TWEAK), and thymic stromal lymphoprotein (TSLP)^13^.

Thrombin, or coagulation factor IIa, is produced from prothrombin by factor Xa which is activated by the complex of factor VIIa and tissue factor (TF). In primary human NP culture, thrombin modulated cytokine and chemokine expression, which increased the level of a variety of inflammatory mediators including CXCL1, IL-6, CXCL8, IL-27, and MCP-1. The downstream molecules protein kinase B (AKT) and glycogen synthase kinase 3 (GSK3)α/β are involved in thrombin-induced epidermal growth factor receptor (EGFR) activation and CXCL8 production in NP cells. In degenerated human NP tissue samples, the expression of EGFR positively correlated with the grade of tissue degeneration^14^.

Protease-activated receptor 1 (PAR1), a member of the 7-transmembrane domain G-coupled receptor family, is a receptor for thrombin. The functions of PAR1 are dependent on cell type; for example, PAR1-initiated platelet aggregation or metastases of malignant cells^15^. Although PAR1 has been investigated in a number of cell types, precisely how PAR1 affects IVDs remains unclear.

Thrombin stimulation induces MCP-1 expression in vascular endothelial cells, vascular smooth muscle, and retinal pigment epithelial cells, and it also activates P38 MAPK, NF-κB signaling^16–19^. We have reported that MCP-1 induced by thrombin treatment via the PI3K/AKT and MAPK-ERK pathways in the fracture healing process, influenced MCP-1 macrophages to migrate to the sites of bone fracture^20^.

Although it is known that thrombin induces cytokine production in IVDs^14^, the precise function and mechanisms underpinning cell migration and IVD degeneration are still poorly understood. The purpose of the current study was to investigate the mechanisms for MCP-1 and MMP-3 induction after treatment with thrombin using coccygeal murine intervertebral disc (mIVD) tissue culture. Our aim is to explore whether thrombin/PAR1 signaling contributes to IVD degeneration.

## Results

### mIVD expression of thrombin and TF

**Figure 1:**
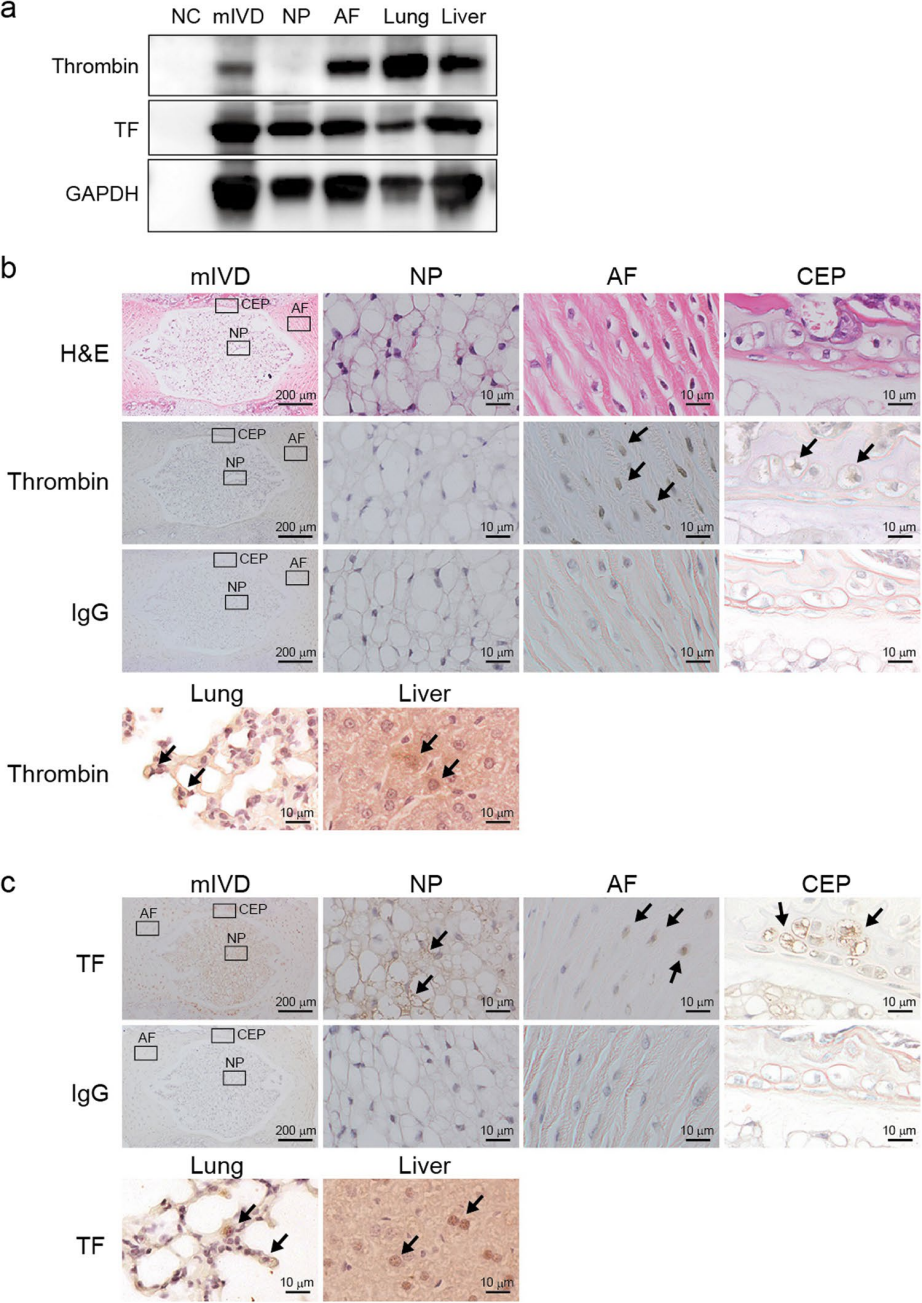
mIVDs expressed thrombin and TF. (**a**) Western blotting analysis showed that thrombin and TF were expressed in mIVDs. AF cells produced not only thrombin but also TF, whereas NP cells produced TF only. NCs were loaded as a negative control and murine lung and liver proteins were loaded as a positive control. GAPDH was used as an internal control. (**b**,**c**) Immunohistological analysis of thrombin and TF protein (brown) expression in NP, AF, and CEPs at high magnification (right) and in whole mIVDs at low magnification (left). No positive cell staining was observed in control Ig-treated sections. Lung and liver tissues were stained with anti-thrombin or anti-TF Ab as a positive control. Representative images from 3 independent experiments are shown (arrow, positive cell; scale bar, 200 or 10 μm). Abbreviations: AF, annulus fibrosis; CEP, cartilage endplate; mIVD, murine intervertebral disc; NCs, no cells; NP, nucleus pulposus; TF, tissue factor. See Supplementary Fig. S1 for examples of uncropped images for each antibody.

Western blotting analysis showed that thrombin and TF were expressed by mIVDs (Fig. 1a). However, AF cells expressed not only thrombin, but also TF, whereas NP cells expressed TF only. No cells (NCs) were loaded as a negative control and protein from lung and liver tissue was loaded as a positive control. Subsequent examinations using IHC staining for thrombin and TF revealed thrombin was localized to the cytoplasm of AF and CEP cells in mIVDs. In contrast, TF was localized to the surface of NP, AF, and CEP cells. TF protein was expressed in NP and AF tissues. Both thrombin and TF were localized to the surface of lung and liver cells that served as positive controls. No positive cell staining was observed in the control Ig-treated sections (Fig. 1b,c).

### mIVD expression of PAR1 thrombin receptors

**Figure 2:**
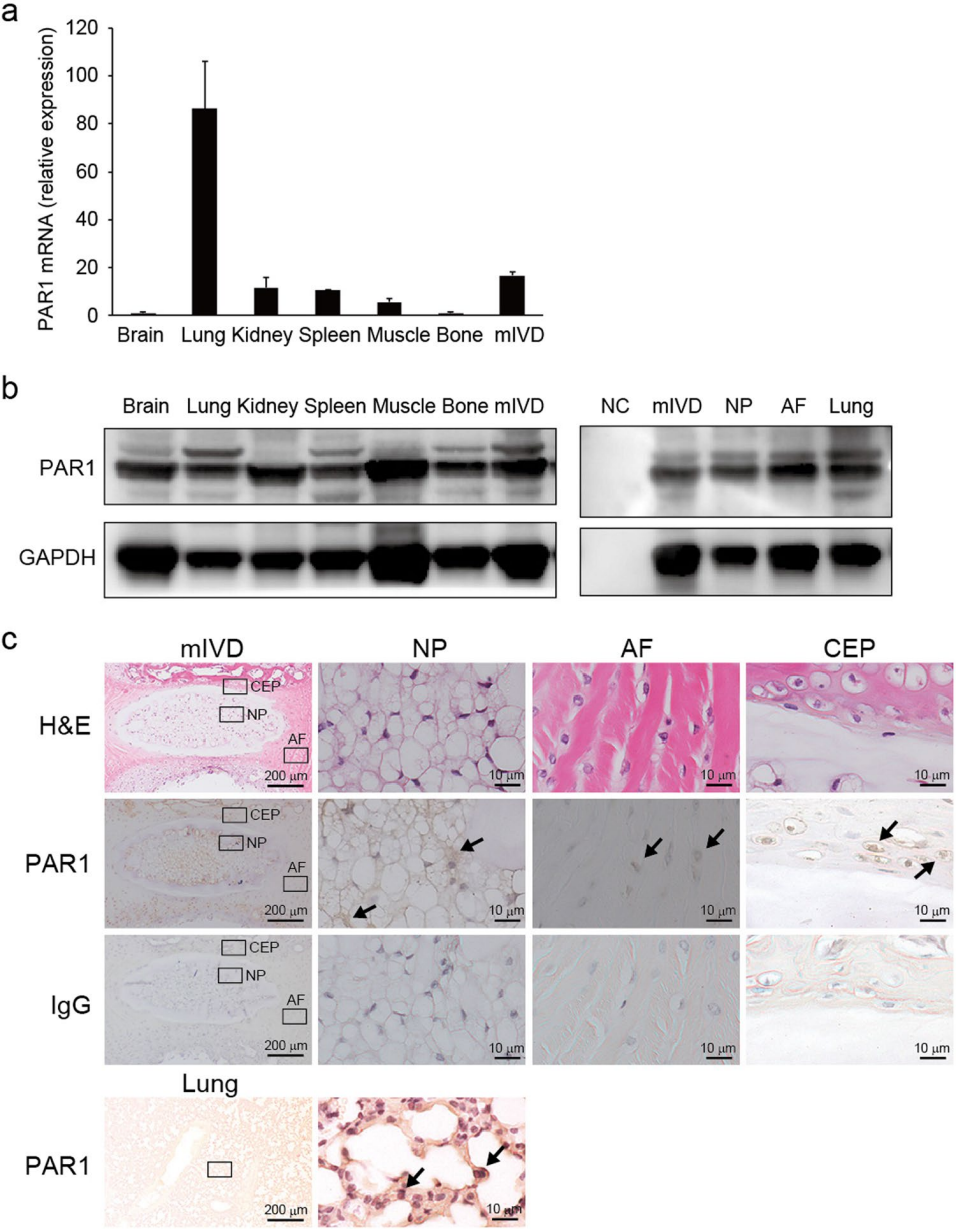
mIVD expression of PAR1 thrombin receptors. (**a**) Tissue was obtained from C57BL/6 mice for RNA and protein extraction. Quantitative PCR was performed using specific primers for *Par1* and *Hprt*. The ratio of each gene compared with that of *Hprt* was calculated, and the value of 1 was assigned to brain tissue. (**b**) Western blotting analysis showed that PAR1 was sufficiently expressed in organ tissues including NP, AF, and total mIVD. NCs were loaded for the negative control and murine lung protein was loaded as a positive control. GAPDH was used as an internal control. (**c**) Immunohistological analysis of PAR1 (brown) expression in NP, AF and CEP (right) at high magnification and in whole mIVDs (left) at low magnification. No positive cell staining was observed in the control Ig-treated sections. Lung tissues were stained with anti-PAR1 Ab for the positive control. Representative images from 3 independent experiments are shown (arrow, positive cell; scale bar, 200 or 10 μm). Abbreviations: AF, annulus fibrosis; CEP, cartilage endplate; GAPDH, glyceraldehyde 3-phosphate dehydrogenase; mIVD, murine intervertebral disc; NCs, no cells; NP, nucleus pulposus; TF, tissue factor. See Supplementary Fig. S2 for examples of uncropped images for each antibody.

*Par1* was expressed in various tissues, including mIVDs; relatively high levels were detected in lung mIVDs (Fig. 2a). PAR1 was expressed in organ tissues including in NP, AF, and total mIVDs. NCs were loaded as a negative control and protein from lung tissue was loaded as a positive control (Fig. 2b). PAR1 in mIVDs was localized to the membranes of NP, AF, and CEP cells as well as lung tissues which served as a positive control. No positive cell staining was observed in the control Ig-treated sections (Fig. 2c).

### Induction of MCP-1 in mIVD via thrombin/PAR1 signaling

**Figure 3:**
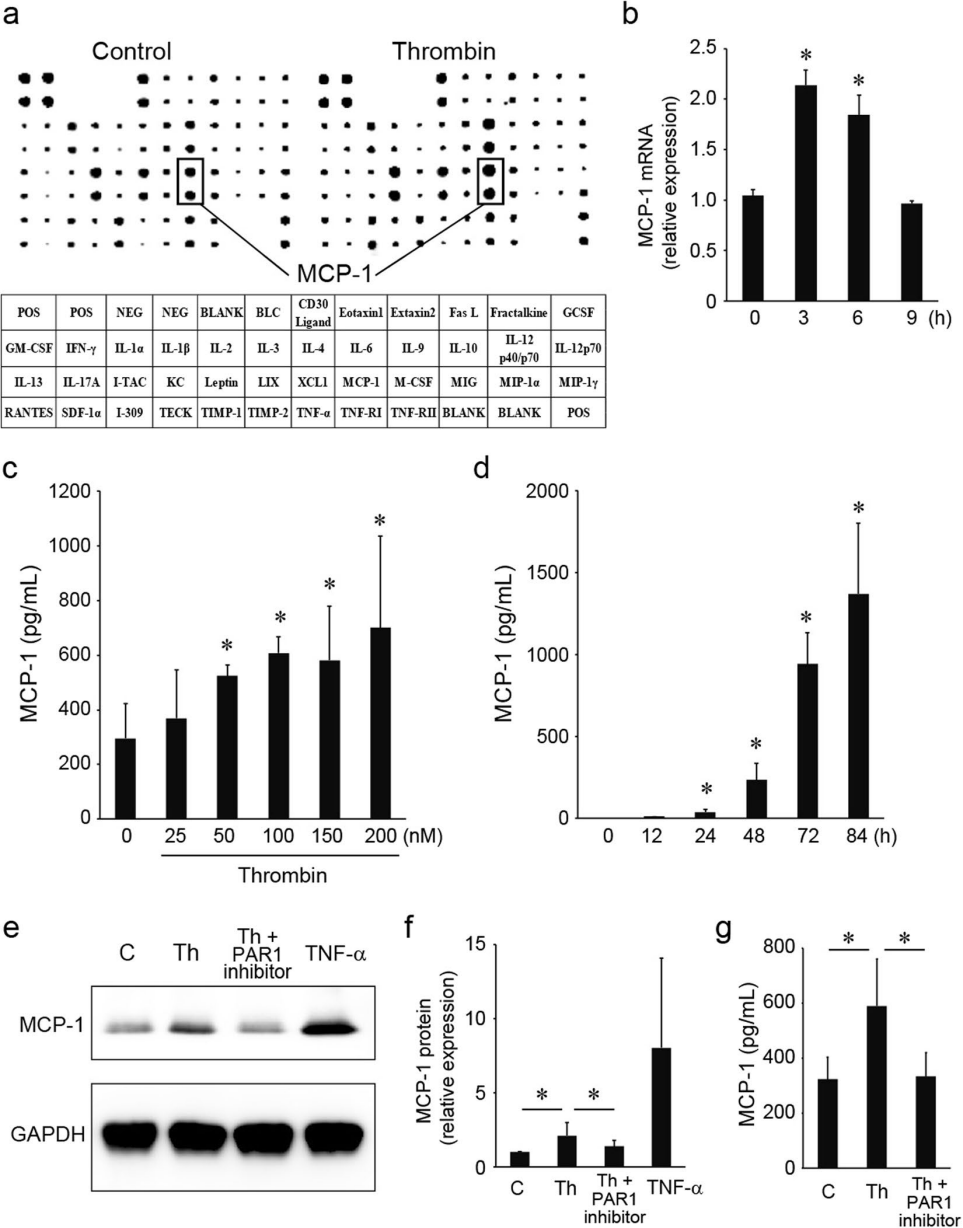
Induction of MCP-1 in mIVD via thrombin/PAR1 signaling. (**a**) mIVDs were cultured in the presence or absence of 100 nM of thrombin for 72 hours. The culture supernatants were collected and subjected to a cytokine protein array. The table indicates the corresponding cytokines on the protein array membrane. (**b**) mIVDs were stimulated with thrombin and quantitative PCR was performed using specific primers for *Mcp-1* and *Hprt*. The ratio of each gene to that of *Hprt* was calculated, and the value of 1 was assigned to no treatment. (**c**,**d**) mIVDs were stimulated with thrombin and culture supernatants were collected to measure the concentration of MCP-1 using an ELISA assay. (**e**,**g**) mIVD were stimulated with thrombin (100 nM) with or without a PAR1 inhibitor (1 µg/mL) for 72 hours. The cell lysates and supernatants were subjected to Western blotting analysis with anti-MCP-1 and anti-GAPDH Ab or analyzed using the ELISA system. (**f**) Images of panel (e) were captured using an LAS-4000 camera system and quantified by imageJ software. Values represent the mean ± SD. *p < 0.05 compared with the corresponding control. Similar results were obtained in at least 3 independent experiments. Abbreviations: C, control; GAPDH, glyceraldehyde 3-phosphate dehydrogenase; Th, thrombin. See Supplementary Fig. S3 for examples of uncropped images for each antibody. See Supplementary Fig. S4 for additional experiments that are the same as Fig. 3e–g with another PAR1 inhibitor. (0.3 µM).

Supernatants from mIVDs cultured in the presence or absence of 100 nM of thrombin for 72 h and subjected to a cytokine protein array showed that thrombin increased the expression of MCP-1, IL-6 and I-309 and decreased the expression of B-lymphocyte chemoattractant (BLC), IL-1α, and IL-13 (Fig. 3a). Using quantitative real time PCR, we confirmed that thrombin significantly increased the expression of *Mcp-1* mRNA in mIVD (Fig. 3b). MCP-1 was increased by thrombin stimulation in a dose- and time-dependent manner (Fig. 3c,d). mIVD stimulated with thrombin (100 nM) with or without a PAR1 inhibitor (1 µg/mL) for 72 h revealed that thrombin-induced MCP-1 production was abrogated by the addition of a PAR1 inhibitor as determined by Western blotting analysis (Fig. 3e,f) and ELISA analysis (Fig. 3g). TNF-α (10 ng/mL) treatment was used as positive control. Additional experiments with another PAR1 inhibitor (0.3 µM) yielded results similar to those shown in Fig. 3e–g and Supplemental Fig. 4.

### MCP-1 produced in mIVD induced macrophage migration

**Figure 4:**
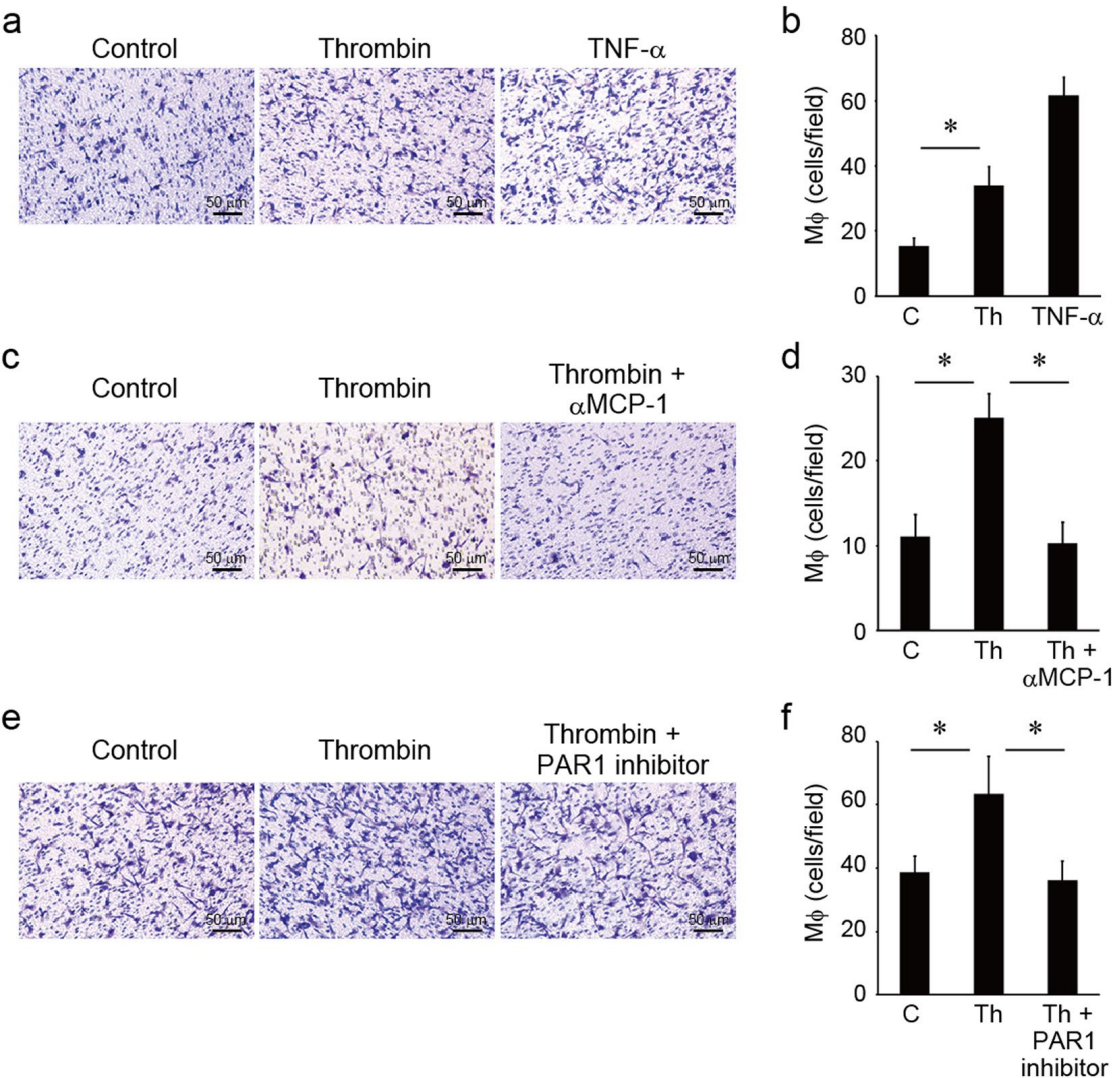
MCP-1 produced in mIVDs induced macrophage migration. (**a**–**f**) mIVDs were cultured in the presence or absence of 100 nM of thrombin and with or without anti-MCP-1 neutralizing Ab (2 µg/mL) or PAR1 inhibitor (1 µg/mL) for 72 h. The culture supernatants were collected and poured to the lower Chemotaxicell chamber. Upper wells were populated with 3 × 10^5^ macrophages in 500 µL of DMEM containing 0.1% FBS. After incubation for 6 hours at 37 °C, cells that had migrated to the lower surface were fixed and stained with crystal violet (scale bar, 50 μm). (**b**,**d**,**f**) The cells on the lower chamber surface were counted in 8–10 fields under high-power magnification (200×). TNF-α (10 ng/mL) treatment was used as positive control. *p < 0.05 compared with the corresponding control. Similar results were obtained in at least 3 independent experiments. Abbreviations: C, control; Th, thrombin.

mIVDs were cultured in the presence or absence of 100 nM of thrombin for 72 h. The culture supernatants were collected and poured to the lower Chemotaxicell chamber. Upper wells were populated with 3 × 10^5^ macrophages in 500 µL of DMEM containing 0.1% FBS. Then these chambers were incubated for 6 h at 37 °C. The MCP-1 produced in mIVDs induced macrophage migration (Fig. 4a,b). TNF-α (10 ng/mL) treatment was used as positive control. Also, anti-MCP-1 neutralizing Ab (2 µg/mL) and PAR1 inhibitor (1 µg/mL) inhibited macrophage migration (Fig. 4c–f).

### Thrombin-induced MCP-1 production in mIVD via PI3K/AKT and MAPK-ERK pathways

**Figure 5:**
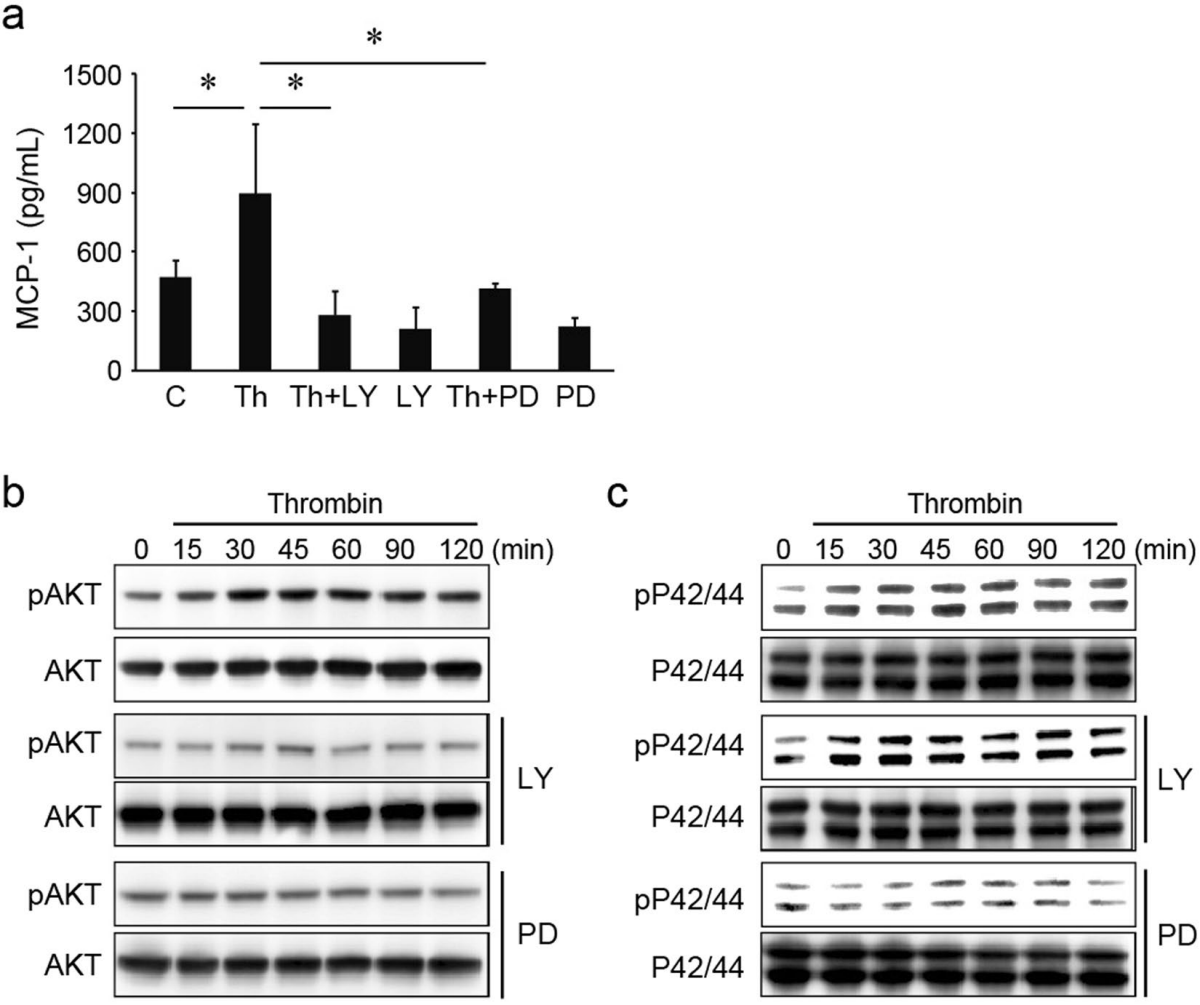
Induction of MCP-1 in mIVDs via thrombin/PAR1 signaling. (**a**) mIVDs were stimulated with thrombin (100 nM) with or without the PI3K inhibitor LY294002 (1 µM) and the inhibitor of MAPK-ERK, PD98059 (1 µM), for 72 hours. The supernatants were analyzed using an ELISA system. (**b**,**c**) mIVDs were stimulated with thrombin in the presence or absence of LY294002 and PD98059. The cell lysates were subjected to Western blotting analysis with Abs specific for phosphorylated AKT, AKT, phosphorylated ERK P42/44, and ERK P42/44. Values represent the mean ± SD. *p < 0.05 compared with the corresponding control. Similar results were obtained in at least 3 independent experiments. Abbreviations: C, control; LY, LY294002; PD, PD98059; Th, thrombin. See Supplementary Figs S5–8 showing uncropped images for each antibody.

mIVDs were stimulated with thrombin (100 nM) with or without the PI3K inhibitor LY294002 (1 µM) and the inhibitor of MAPK-ERK, PD98059 (1 µM), for 72 hours. The supernatants were analyzed using an ELISA system. LY294002 and PD98059 significantly suppressed thrombin-induced MCP-1 production in mIVDs (Fig. 5a); however, it was not abrogated by the addition of SB203580 (data not shown). Phosphorylation of AKT was induced by treatment of mIVD with thrombin, but was abrogated by the addition of LY294002 or PD98059 (Fig. 5b). Phosphorylation of ERK p42/44 was also induced by the treatment of mIVD with thrombin, whereas it was suppressed by the addition of PD98059, but not LY294002 (Fig. 5c).

### Induction of MMP-3 in mIVD via thrombin/PAR1 signaling

**Figure 6:**
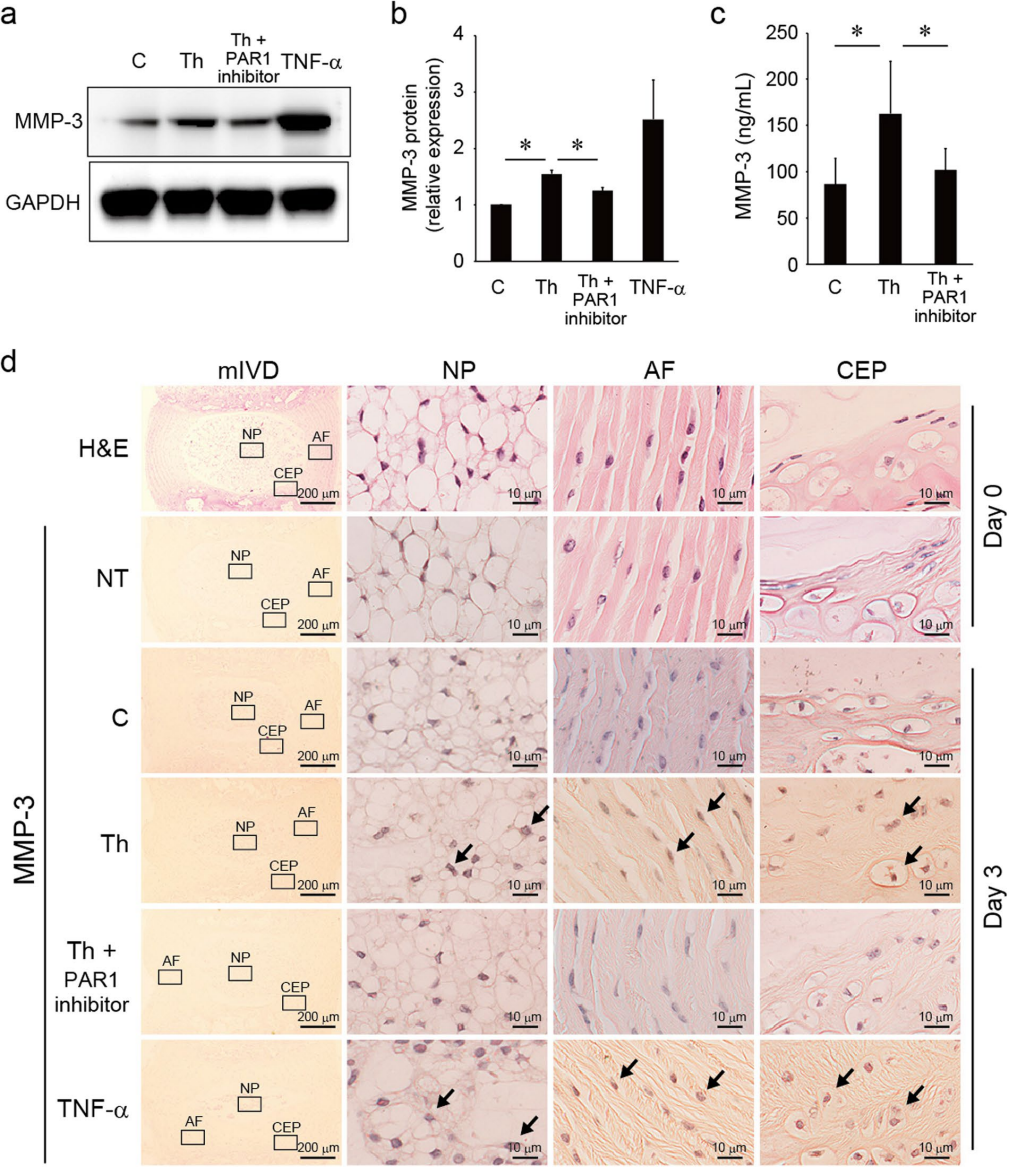
Induction of MMP-3 in mIVDs via thrombin/PAR1 signaling. (**a**,**c**) mIVDs were stimulated with thrombin (100 nM) with or without a PAR1 inhibitor (1 µg/mL) for 72 hours. The cell lysates and supernatants were subjected to Western blotting analysis with anti-MMP-3 and anti-GAPDH Abs or analyzed using the ELISA system. GAPDH was loaded as a control. (**b**) Images of Fig. 6a were captured using an LAS-4000 camera system and quantified by imageJ software. Values represent the mean ± SD. *p < 0.05 compared with the corresponding control. Similar results were obtained in at least 3 independent experiments. (**d**) mIVDs were stimulated with thrombin with or without PAR1 inhibitor for 72 hours. Immunohistological analyses were performed for MMP-3 (brown) expression in NP, AF, and CEPs (right) at high magnification and in whole mIVDs (left) at low magnification. No positive cell staining was observed in the untreated and control sections. TNF-α-treated tissues were stained with anti-MMP-3 Ab as a positive control. Representative images from 3 independent experiments are shown (arrow, positive cell; scale bar, 200 or 10 μm). Abbreviations: AF, annulus fibrosis; C, control; CEP, cartilage endplate; GAPDH, glyceraldehyde 3-phosphate dehydrogenase; mIVD, murine intervertebral disc; NP, nucleus pulposus; NT, not treated; Th, thrombin. See Supplementary Fig. S9 for examples of uncropped images for each antibody.

mIVDs were stimulated with thrombin (100 nM) with or without a PAR1 inhibitor (1 µg/mL) for 72 hours. This study confirmed that thrombin in mIVDs significantly increased the production of MMP-3. Thrombin-induced MMP-3 was abrogated by the addition of a PAR1 inhibitor as was determined by Western blotting analysis (Fig. 6a,b) and ELISA analysis (Fig. 6c). In mIVD, MMP-3 was localized to the cytoplasm of NP, AF, and CEPs. Thrombin increased the numbers of cells positive for MMP-3, an effect abrogated by the addition of a PAR1 inhibitor (Fig. 6d). No positive cell staining was observed in the untreated and control sections. TNF-α (10 ng/mL) treatment was used as positive control.

### Treatment of mIVD with thrombin induced disc degeneration

**Figure 7:**
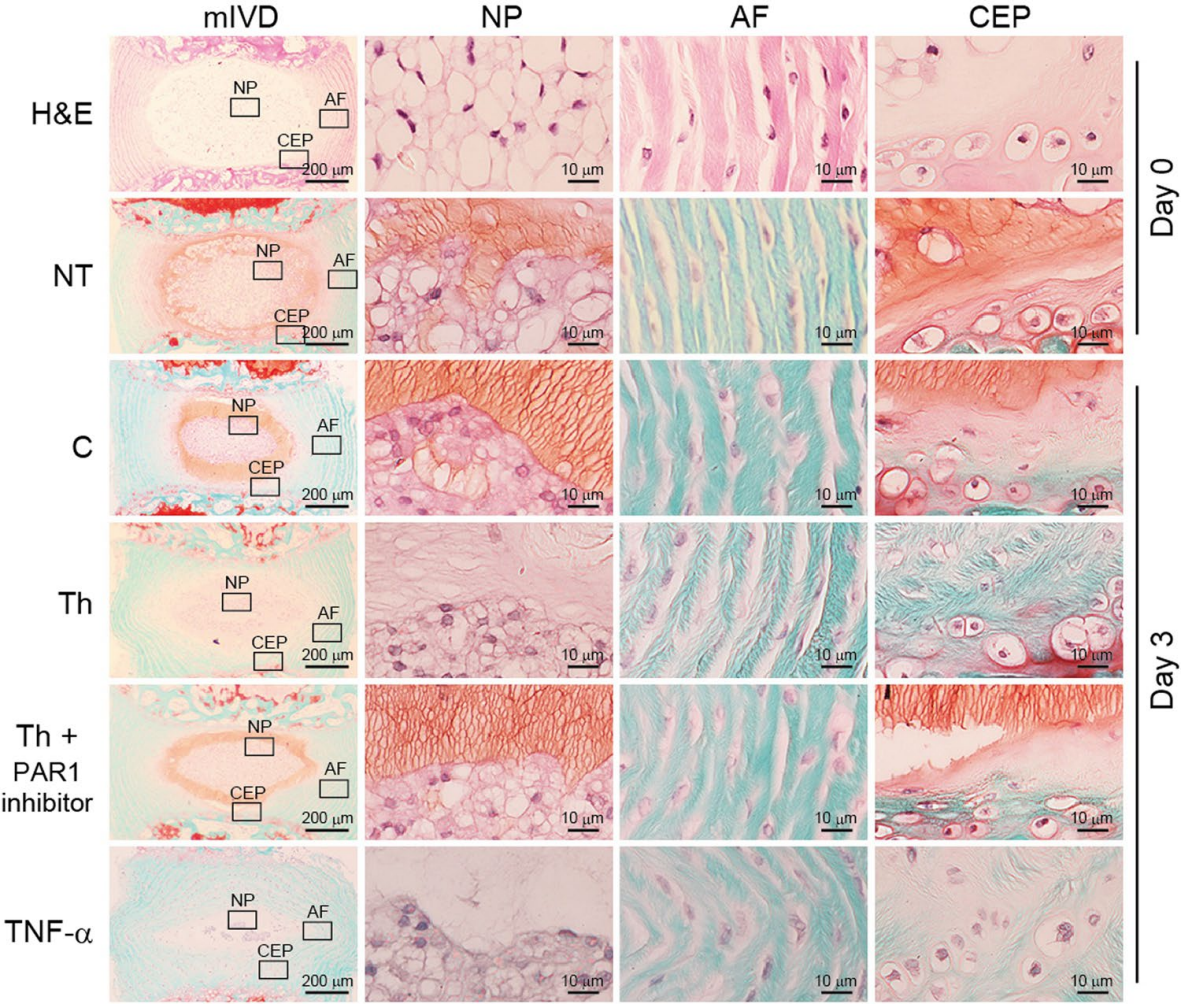
Treatment of mIVDs with thrombin induced disc degeneration. mIVDs were stimulated with thrombin (100 nM) in the absence or presence of a PAR1 inhibitor (1 µg/mL) for 72 hours. Safranin-O and fast green stains were performed for proteoglycan expression (orange) in NP, AF, and CEPs at high magnification (right) and in whole mIVDs (left) at low magnification. TNF-α-treated tissues were stained with Safranin-O and fast green as a positive control for mIVD degeneration. Representative images from 3 independent experiments are shown (scale bar, 200 or 10 μm). Abbreviations: AF, annulus fibrosis; C, control; CEP, cartilage endplate; GAPDH, glyceraldehyde 3-phosphate dehydrogenase; mIVD, murine intervertebral disc; NP, nucleus pulposus; NT, not treated; Th, thrombin.

Under nonstimulatory conditions, Safranin-O staining of aggrecan was observed around NP cells and CEP cells. Degradation of mIVDs in the presence of thrombin was confirmed by the loss of Safranin-O staining after 72 h of culturing, a phenomenon abrogated by the addition of a PAR1 inhibitor (1 µg/mL) (Fig. 7), which thus demonstrated that the administration of thrombin degraded proteoglycan via PAR1. Safranin-O staining of TNF-α (10 ng/mL) treatment was used as positive control of degradation.

## Discussion

Intervertebral disc degeneration is thought to be caused by NP cells themselves or environmental changes^5^, whereas almost no structural change is observed in the AF. Degeneration results in dehydration, a decreasing amount of proteoglycan, and a reduction in the height of IVDs. Disc herniation in the spine occurs when the AF tears and the production of inflammatory cytokines causes progressive disc degeneration^2,3,21^. Cytokine-induced degeneration occurs via various pathways. In the current study, the focus was on thrombin, which is one of the initiators of inflammation, and therefore, its relationship with MCP-1 was examined.

Although a low concentration of thrombin can be tolerated in response to various injuries, a high concentration of thrombin induces cell death in the central and peripheral nervous systems. Thrombin is involved in mediating neuronal cell death in cerebral ischemia via PAR1^14,22–24^. In this study, thrombin was endogenously produced in mIVDs and localized to the cytoplasm of the AF and CEPs, but was not present in the NP (Fig. 1a,b). In the blood coagulation pathway, TF, also referred to as factor III, complexes with factor VIIa and activates factor X to Xa, and then Xa activates prothrombin to thrombin^15^. Our results confirm that TF was expressed in mIVDs, especially in the NP, AF, and CEPs (Fig. 1a,c). These data suggest that the presence of TF promotes thrombin-induction localized to mIVDs. Although NP cells expressed TF, there was no expression of thrombin. This result appears to be related to the avascular nature of the NP. Our previous reports also suggest that thrombin may be produced in NP cells concurrently with angiogenesis during aging, for the maintenance of homeostasis^11,25^.

PAR1 is expressed in the musculoskeletal system by osteoblasts, chondroblasts, and myoblasts^26^. Knockout mouse models characterized by prothrombin, TF, or *Par1* deletions demonstrate embryonic lethality^27,28^. Thrombin is reportedly necessary to form yolk sac blood vessels via the activation PAR1^29^, suggesting that PAR1 may play a particularly important role in angiogenesis around IVDs during the developmental stage. In contrast, angiogenesis can be observed during the late phase of degeneration and result in the decreased activity of NP cells^9^. Our study confirmed that PAR1 was expressed in many murine tissues including IVDs, NP, AF, and CEPs (Fig. 2a–c).

MCP-1 expression is mediated by various stimuli including mechanical and oxidative stress^30^. MCP-1 plays critical roles in atherosclerosis, rheumatoid arthritis, and bone tumor metastasis^31–35^. MCP-1 is one of the key regulators of inflammation via the recruitment of macrophages and mesenchymal stem cells to the sites of inflammation^36^. Thrombin exclusively induced MCP-1 and IL-6 in this study as was determined via a protein array system (Fig. 3a). This result is consistent with the protein array in a previous report using human NP cells which showed thrombin increased the expression of CXCL1, IL-6, CXCL8, IL-27, and MCP-1 and decreased the expression of CD154, IL-16, IL-23, and IL-13^14^. MCP-1 production was increased by thrombin stimulation in a dose- and time-dependent manner in our study, an effect that was abrogated by the addition of a PAR1 inhibitor (Fig. 3b–g). Huang *et al*. showed thrombin-enhanced MCP-1 production in human NP cells. Our study demonstrated that TF and PAR1, but not thrombin, was expressed on NP cells of murine IVD. Our results are consistent with the need for thrombin to be present, or angiogenesis to be evident around an NP, before MCP-1 can be produced from NP cells.

We have previously reported that TNF-α-induced expression of MCP-1 in herniated discs facilitates the resorption of herniated disc tissue via macrophage recruitment^13^. We confirmed that the MCP-1 produced in mIVDs induced macrophage migration which was abrogated by the addition of anti-MCP-1 neutralizing Ab (Fig. 4a–d). Macrophages migrate to damaged tissue and differentiate into M1/M2 phenotypes in response to the local environment. Differentiation into the M1 phenotype occurs in response to granulocyte-macrophage-CSF, IFNγ, MCP-1, TNF-α and IL-1β synthesized during acute inflammation and M1 digestion of the extracellular matrix occurs in the IVD^30,37–40^. Endogenous production of MCP-1 in IVDs localizes in the cytoplasm of NP and AF cells^41^. It is suggested that in the acute phase of trauma-induced inflammation or IVD herniation, increased thrombin concentrations in AF cells induce MCP-1, causing macrophage migration. In turn, AF and macrophage interaction may cause changes in macrophage phenotype that subsequently induce structural changes in the outer region of AF cells.

Several pathways which implicate P38, P42/44, NF-κB, PI3/AKT, and JNK in MCP-1 expression in other cells (vascular endothelial cells, vascular smooth muscle cells, retinal pigment epithelial cells, and osteoblasts) in mouse, rat, or human have been reported^16–18,20^. This study demonstrated that treatment of mIVDs with thrombin induced PI3K/AKT and MAPK-ERK P42/44 signaling and resulted in cytokine production. We investigated which signaling processes were upstream. Both the phosphorylation of AKT and the phosphorylation of ERK P42/44 induced by thrombin treatment were abrogated by the addition of the MAPK inhibitor PD98059. However, the PI3K inhibitor LY294002 decreased the phosphorylation of AKT, but did not affect the phosphorylation level of ERK P42/44 (Fig. 5a–c). These results indicate that when mIVD was activated by thrombin, MAPK-ERK signaling was probably upstream from PI3K/AKT signaling. It has been reported that the PI3K/AKT pathway in IVDs holds the extracellular matrix construct resulting in MCP-1-induced inflammatory reactions mediated by M1 macrophages^13,42–44^.

Following the acute phase inflammatory response in IVDs, MMPs are abundantly synthesized, and the MMP-3 generated directly degrades proteoglycan, laminas, and components of the IVD extracellular matrix. MMP-3 also activates other MMPs as an indirect action^45,46^. Moreover, MMP-3 changes the population of NP cells^2^ causing macrophage migration during disc herniation^10,11^. In the current study, MMP-3 was secreted from mIVDs via PAR1 in response to treatment with thrombin (Fig. 6) suggesting that MMP-3 may be involved in disc degeneration via proteoglycan degeneration and changes in the population of NP cells. mIVD was degraded in the presence of thrombin, as confirmed by loss of Safranin-O staining^47^, and abrogated by the addition of a PAR1 inhibitor (Fig. 7). These data suggest that thrombin degraded proteoglycan via PAR1 and that PAR1-induced MMP-3 production might be involved in disc degeneration.

This study has some limitations. First, we used only mIVDs. Additional studies that utilize the IVDs of other animal models including rats, rabbits, or pigs could further elucidate the mechanism of thrombin-regulated disc degeneration. Second, we cultured mIVDs for only 6 to 84 hours in identical culture conditions; however, variations in the culture medium, culture plate, degree of cell confluence, oxygen concentration, and/or carbondioxide concentration could produce different results. Third, although we showed disc degeneration via MMP-3 or thrombin directly was possible, this study did not investigate the presence of thrombin-induced macrophages as a result of mIVD degeneration.

In conclusion, we demonstrated a pivotal role for thrombin in mIVD degeneration. TF and PAR1 are endogenously expressed in mIVDs. Thrombin/PAR1 signaling in mIVDs regulated MCP-1 and MMP-3 production via the MAPK-ERK and PI3/AKT pathways and MCP-1 enhanced macrophage migration. Thus, thrombin may be a novel factor capable of stimulating the cytokine activity that regulates several aspects of mIVDs. Observations from this study contribute to a better understanding of the degenerative and homeostatic mechanisms governing mIVDs which are regulated by thrombin/PAR1 signaling.

## Materials and Methods

### Reagents

Purchased for use in this study was a PAR1 antagonist (YFLLRNP) from AnaSpec, Inc. (OH, Fremont, CA, USA), PAR1 inhibitor (SCH79797) from Santa Cruz Biotechnology, Inc. (TX, USA), a PI3K inhibitor (LY294002) obtained from Cayman Chemical (Ann Arbor, MI, USA), a MAPK-ERK inhibitor (PD98059), a P38 inhibitor (SB203580) from Merck KGaA (Darmstadt, Germany), and mouse MCP-1 Ab from R&D Systems (Minneapolis, MN, USA).

### Animals

Homozygous WT C57BL/6J mice (5–6 weeks old) were purchased from CLEA Japan, Inc., or Japan SLC, Inc., (Japan). The mice were housed at 22–24 °C with a 12-h light/dark cycle and maintained on standard mouse chow and water provided *ad libitum*.

All experiments with mice were conducted according to the Guidelines for Proper Conduct of Animal Experiments, Science Council of Japan, and protocols were approved by the Animal Care and Use Committee (No. 17–11), University of Yamanashi.

### Murine intervertebral disc organ culture

A mIVD organ culture system was prepared as previously described^11^. In brief, mIVD tissue specimens were obtained from the tail bone of mice using a dissecting microscope after the skin and soft tissue were removed. Whole mIVD tissue specimens were cultured in 12-well plates in 1 mL of DMEM sourced from Invitrogen/Gibco (Carlsbad, CA, USA) containing 0.1% FBS in the presence or absence of the indicated doses of thrombin or mouse TNF-α (10 ng/mL), obtained from R&D Systems (Minneapolis, MN, USA), at 37 °C.

### Western blot analysis

mIVD tissues (9 discs) were collected and a protein assay was performed to quantitate NP and AF using a CelLytic MT cell lysis reagent (Sigma) according to the manufacturer’s instructions (St. Louis, MO, USA). Equal amounts of protein from each sample were analyzed as previously described^48^ by immunoblotting with primary Abs against thrombin, PAR1, and MMP-3 obtained from Santa Cruz Biotechnology, Inc. (TX, USA) and mouse TF and another MMP-3 from Abcam (Cambridge, MA, USA). Also, mouse specific Ab MCP-1, glyceraldehyde 3-phosphate dehydrogenase, phospho-AKT (Ser473) and AKT Ab, phosphor-P42/44 MAPK (Thr202/Thr204), and P42/44 MAPK Ab sourced from Cell Signaling Technology (MA, USA), were used. Images were captured using a LAS-4000 camera system from Fujifilm (Tokyo, Japan) and were quantified using imageJ software (Wayne Rasband, National Institutes of Health).

### ELISA assay

mIVDs were cultured in 12-well plates. Conditioned media were collected and prepared by microcentrifugation at 5,000 rpm for 5 minutes, twice. Concentrations of MCP-1 and MMP-3 in conditioned media were determined using a Quantikine quantitative colorimetric ELISA according to the manufacturer’s (R&D Systems) specifications. The absorbance was determined using a microplate reader SH-1100R (Lab) from Corona Electric Co., Ltd (Ibaraki, Japan).

### Quantitative real-time PCR

Following the indicated doses and incubation times in culture, mIVD tissues (9 discs, ~10 µg) were collected and total RNA was extracted using an Isogen total RNA assay kit from Nippon Gene (Toyama, Japan) according to the manufacturer’s instructions. Complementary DNA was synthesized from 2 µg of total RNA using the Reverse Transcriptase System from Applied Biosystems (Foster City, CA, USA). Quantitative PCR analysis was performed using the Applied Biosystems ABI 7500 Fast Real-Time PCR System according to the manufacturer’s instructions. Primers and probes for mouse *Par1* (Mm00438851_m1), mouse *Mcp-1* (Mm00441242_m1), and mouse hypoxanthine phosphoribosyl transferase (*Hprt*) (Mm01545399_m1) were also purchased from Applied Biosystems. The ratio of gene expression to *Hprt* expression was calculated, and a relative value of 1.0 was assigned to brain or mIVD tissues that were incubated without thrombin.

### Cytokine protein array

A cytokine protein array was performed as previously described^49^. mIVDs were stimulated with or without thrombin for 72 hours. The amounts of several cytokines in the culture supernatants were determined by using the RayBio Mouse Inflammation Antibody Array C1 (Norcross, GA, USA) according to the manufacturer’s instructions.

### Preparation of macrophages

Murine peritoneal macrophages were harvested by peritoneal lavage 4 days after intraperitoneal administration of 3 mL PBS containing 3% thioglycollate medium from Oxoid Ltd., (Hampshire, England), as previously described^10^.

### Migration assays

Cell migration assays were performed as previously described^13,31^. The migration activity of peritoneal macrophages was examined using a modified Boyden chamber assay or Chemotaxicell (Kurabo, Tokyo, Japan). The upper and lower chambers were separated with filters equipped with 5-µm pores. Three discs in each well of a 24-well plate were cultured in 1 mL of DMEM containing 0.1% FBS in the presence or absence of α-thrombin (100 nM) or TNF-α (10 ng/mL) as a positive control. Supernatants were poured into the lower chambers. Upper wells were populated with 3 × 10^5^ macrophages in 500 µL of DMEM containing 0.1% FBS. After incubation for 6 h at 37 °C, the membrane was removed and washed with PBS and nonmigrated cells on the upper surface of the membrane were removed with a cotton swab. Cells were fixed in 70% ethanol for 5 minutes and stained with Sigma crystal violet solution (MO, USA) for 5 minutes. The cells on the lower surface were counted in 8–10 fields under high-power magnification (200X).

### Histological analysis

Histological analysis was performed as previously described^42^. The coccygeal IVDs of mice were fixed in 4% paraformaldehyde for 3 days, defatted for 3 days, and decalcified with 10% EDTA for 7 days. Discs were paraffin-embedded and consecutive 5-μm sections were stained with H&E or Safranin-O purchased from Merck (Darmstadt, Germany) and fast green from Sigma. Sections of mIVD samples treated with thrombin or recombinant mouse TNF-α were stained with 0.25% Safranin-O as an indicator of proteoglycan content. Immunohistochemical (IHC) staining for thrombin, anti-TF Ab, PAR1, MMP-3, or control IgG, was performed, using the Dako North America Inc., Liquid DAB + Substrate Chromogen System (Carpinteria, CA, USA) according to the manufacturer’s specifications, and counterstained with hematoxylin.

### Statistical analysis

Data are presented as the mean ± SD. Significance was determined using the Student’s or Welch’s *t*-test after an *F*-test was performed, unless otherwise stated. If the raw data did not fit a normal distribution, the Mann-Whitney *U*-test was used. A *p* value of < 0.05 was considered statistically significant.

## Electronic supplementary material

Supplementary Figures
