# Supplementary material for: Effect of Thrombin-Induced MCP-1 and MMP-3 Production Via PAR1 Expression in Murine Intervertebral Discs
Source: Sci Rep. 2018 Jul 27;8:11320. doi: 10.1038/s41598-018-29669-z (PMC6063965; doi:10.1038/s41598-018-29669-z)
Supplement: Supplementary file 1 — Supplementary Figures [file 41598_2018_29669_MOESM1_ESM.pdf]

Supplementary Information

Effect of Thrombin-Induced MCP-1 and MMP-3 Production Via PAR1 Expression in Murine Intervertebral Discs

Yoshihiro Takayama, Takashi Ando*, Jiro Ichikawa, and Hirotaka Haro

Department of Orthopaedic Surgery, Faculty of Medicine, University of Yamanashi, 1110 Shimokato, Chuo, Yamanashi, 409-3898, Japan

Supplementary Figure S1 Examples of Uncropped Western Blots

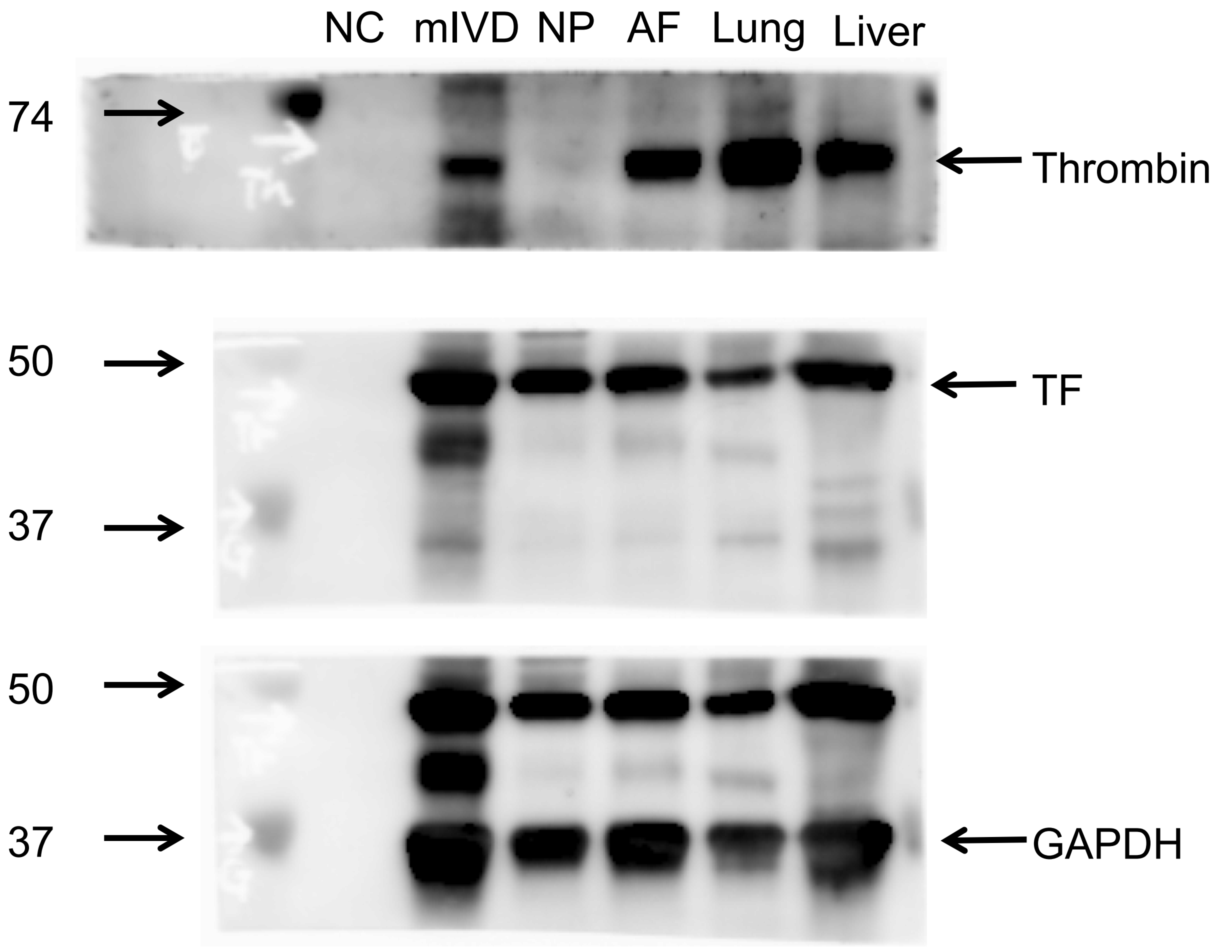

Examples of Uncropped Western Blots for Thrombin, TF, and GAPDH from Figure 1a.

Supplementary Figure S2 Examples of Uncropped Western Blots

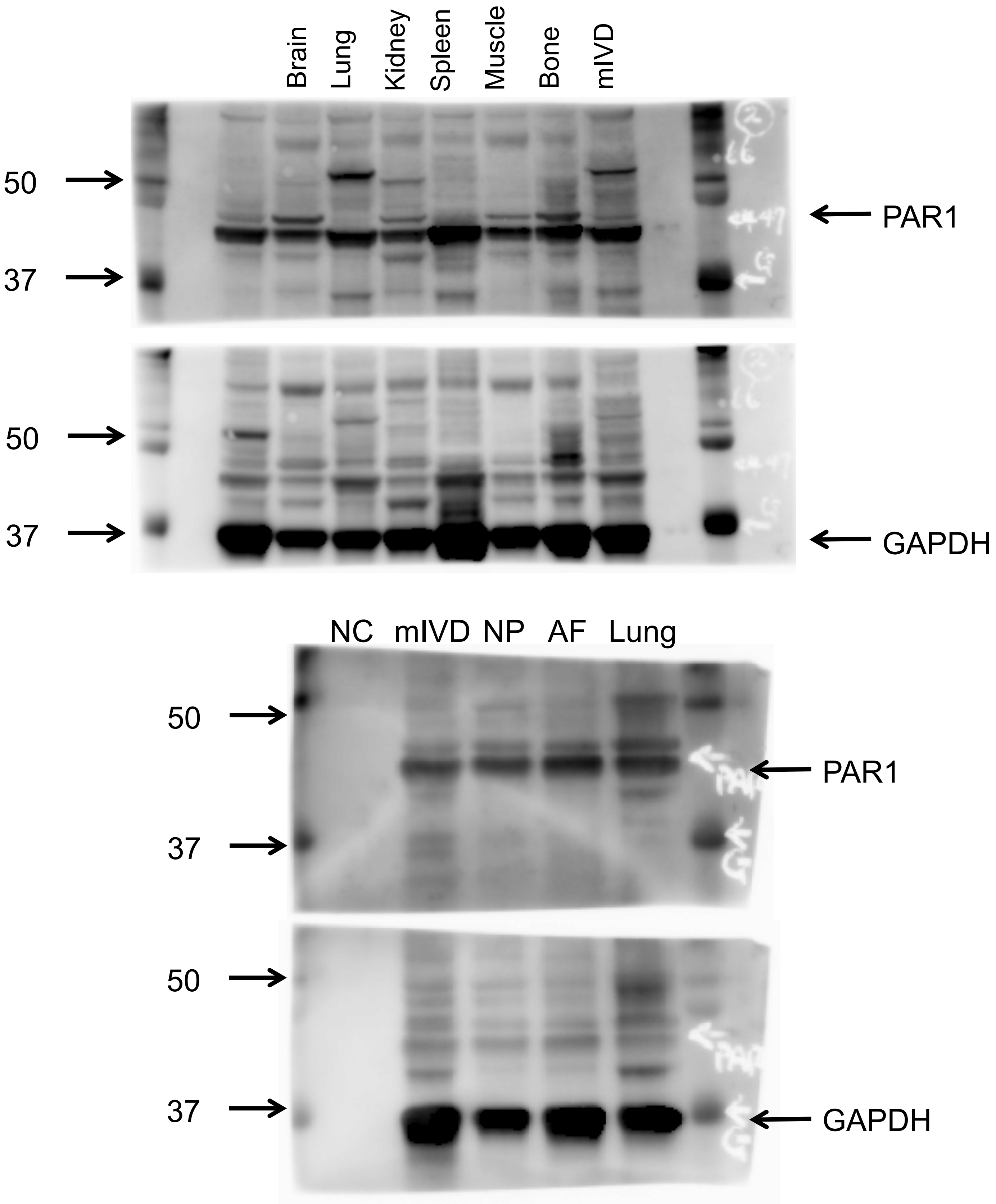

Examples of Uncropped Western Blots for PAR1 and GAPDH from Figure 2b.

Supplementary Figure S3 Examples of Uncropped Western Blots

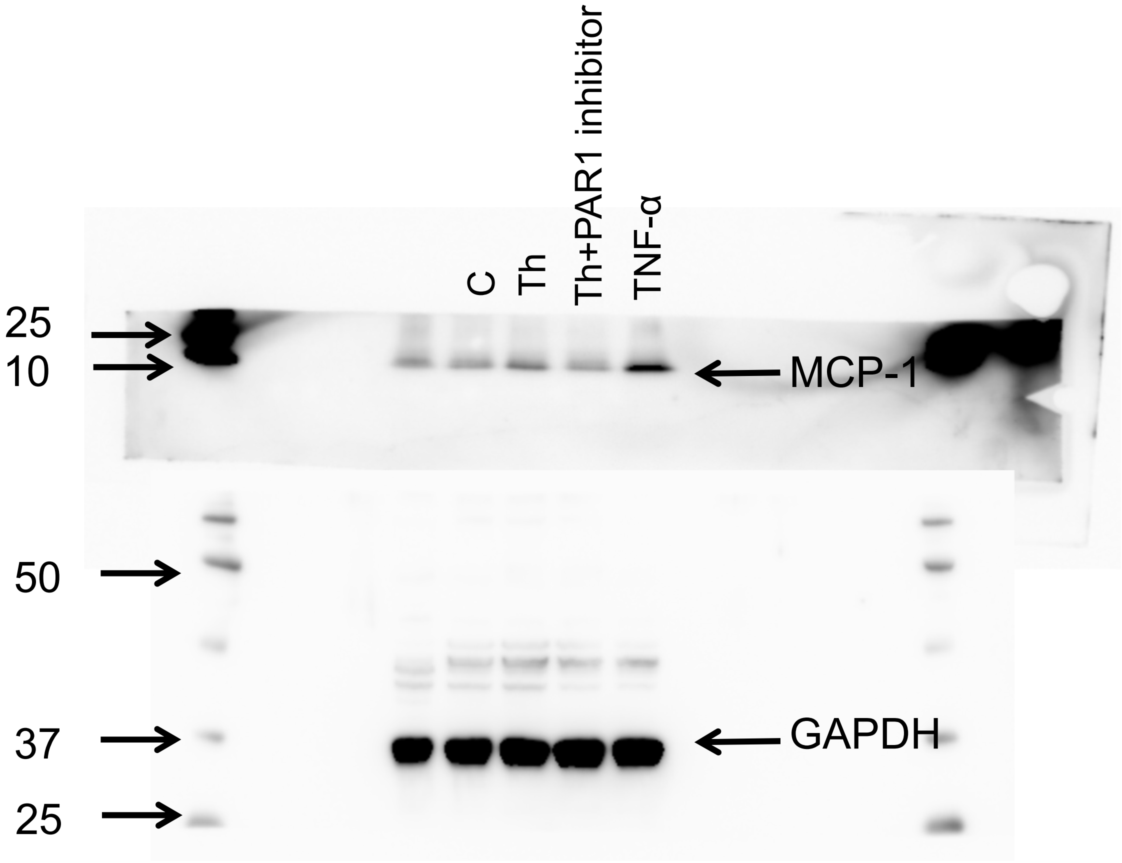

Examples of Uncropped Western Blots for MCP-1 and GAPDH from Figure 3e.

Supplementary Figure S4 Examples of Uncropped Western Blots

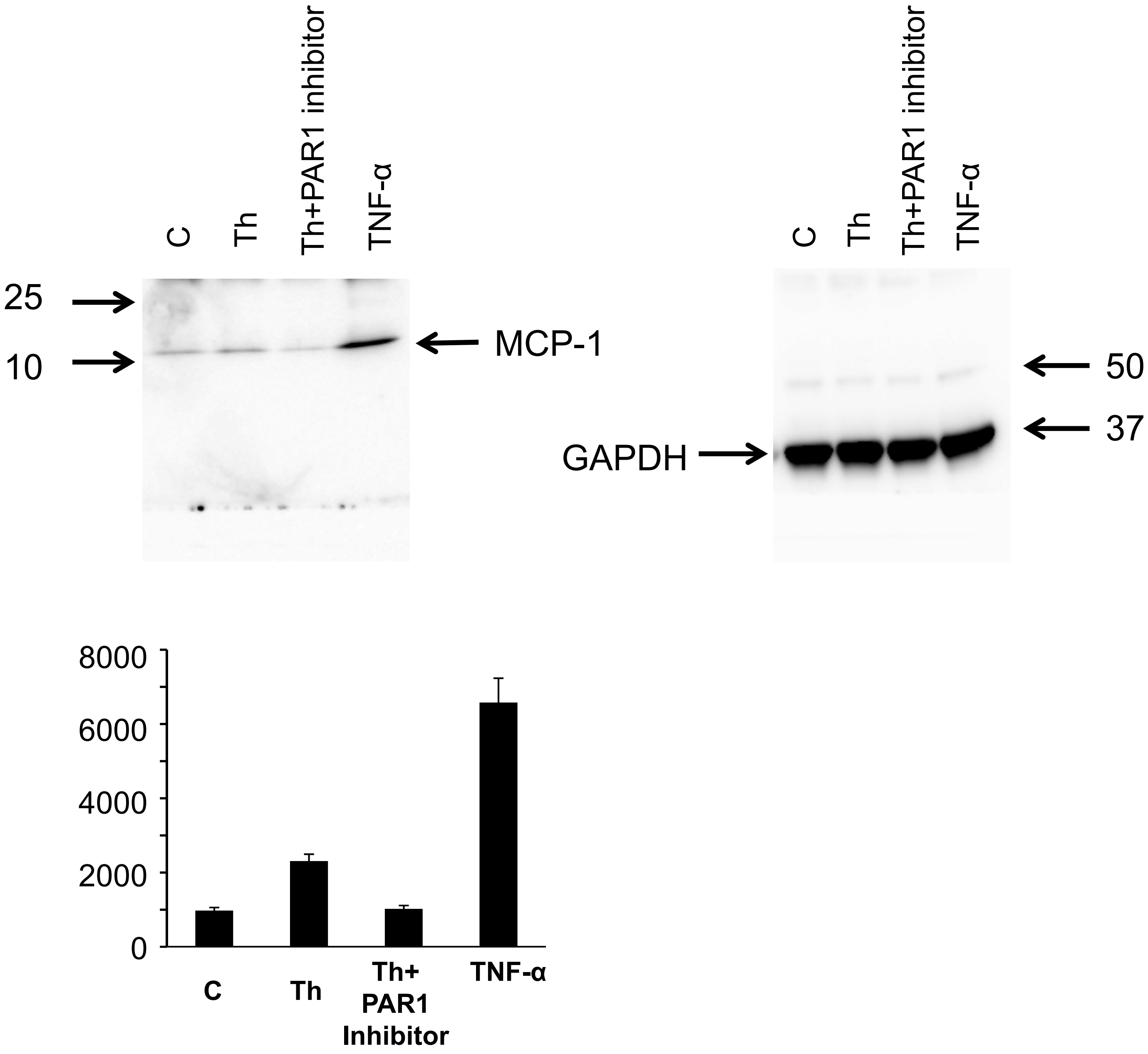

Same as Figure 3e–g. experiments using another PAR1 inhibitor (SCH79797)

Supplementary Figure S5 Examples of Uncropped Western Blots

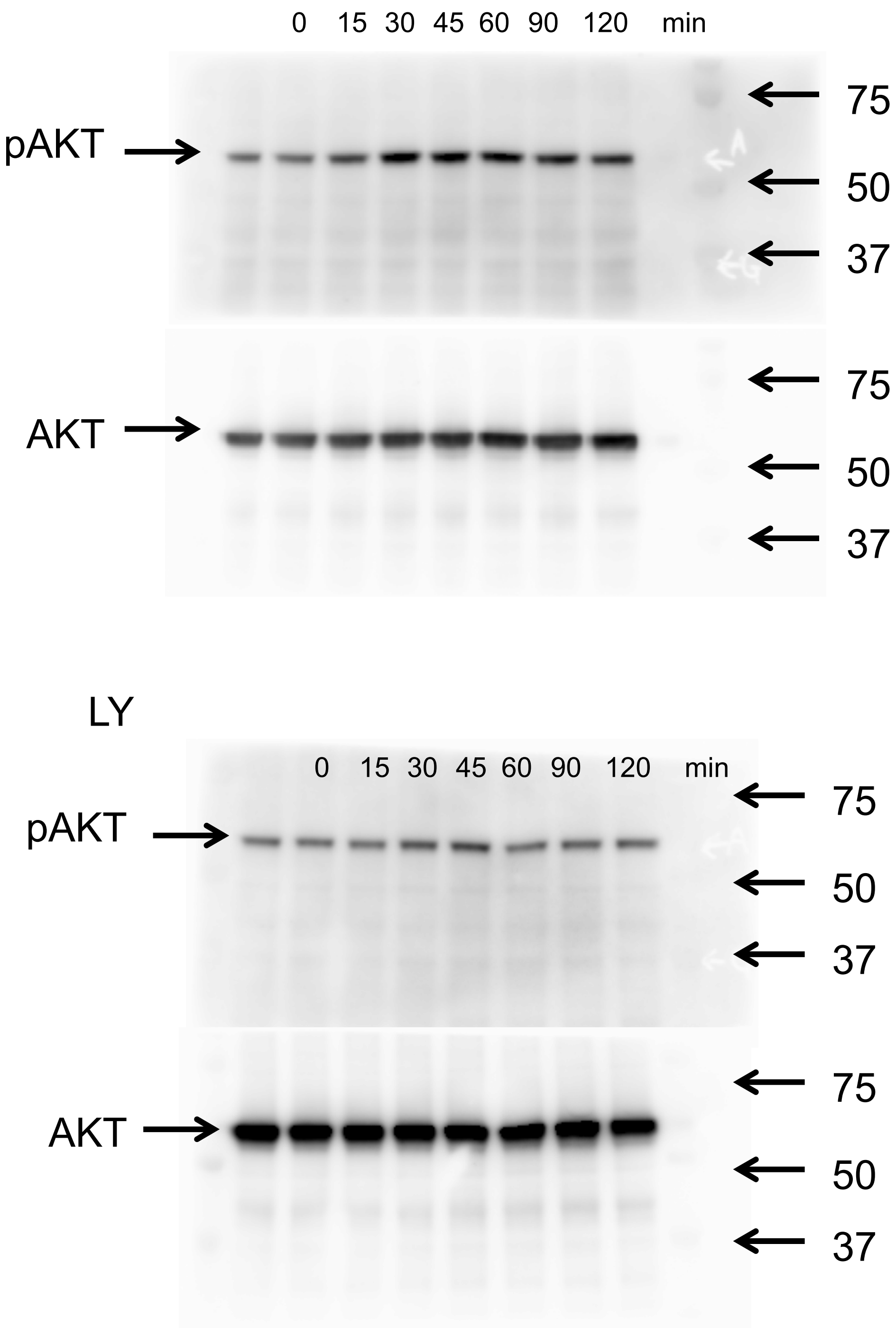

Examples of Uncropped Western Blots for pAKT and AKT
with or without LY294002 from Figure 5b.

Supplementary Figure S6 Examples of Uncropped Western Blots

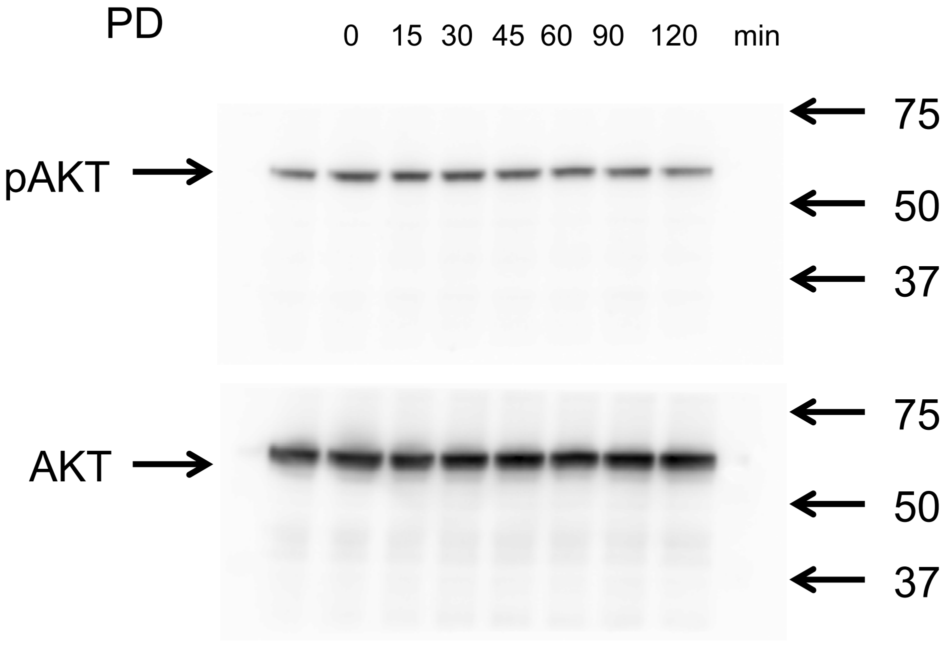

Examples of Uncropped Western Blots for pAKT and AKT with PD98059 from Figure 5b.

Supplementary Figure S7 Examples of Uncropped Western Blots

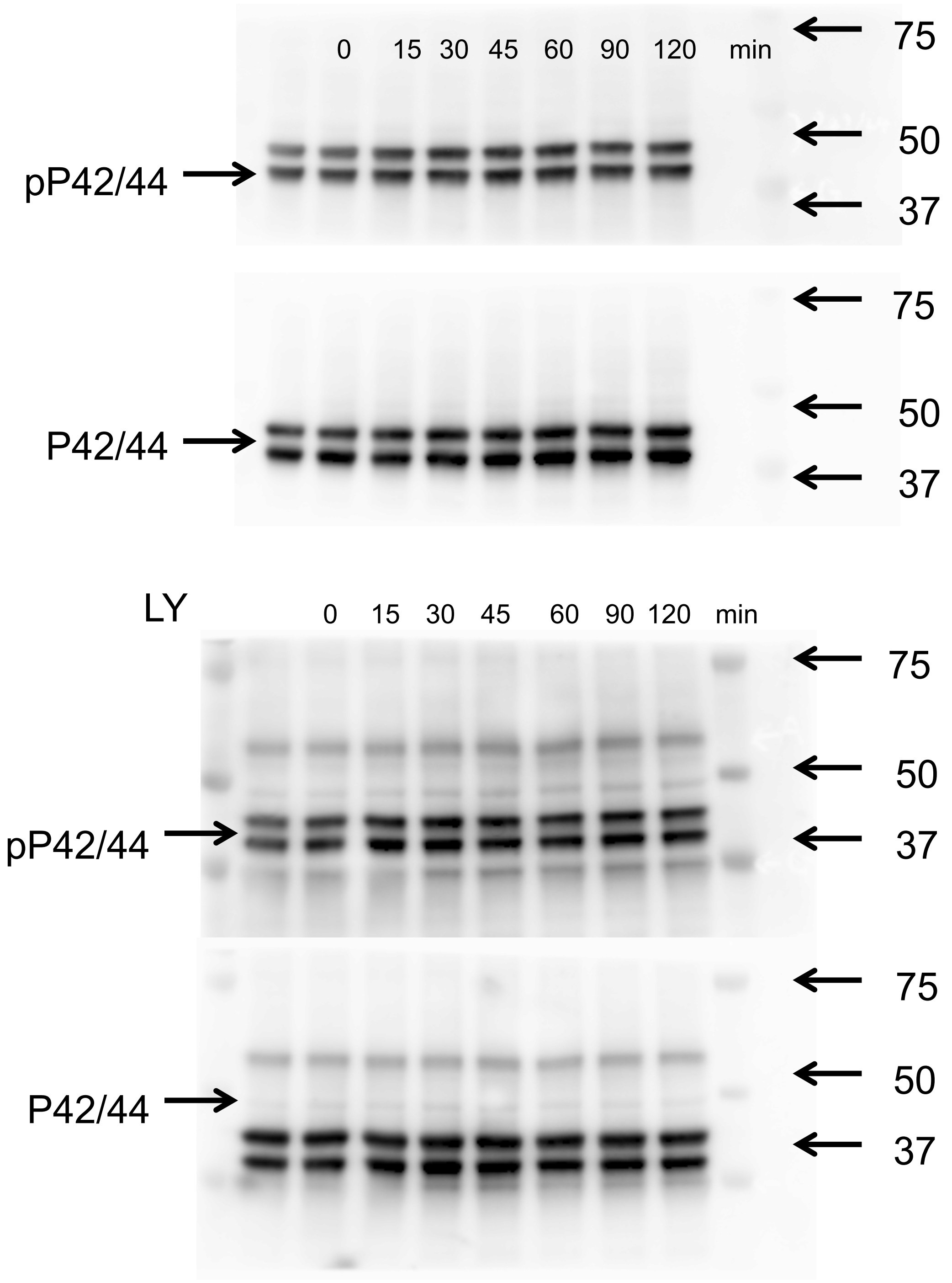

Examples of Uncropped Western Blots for pP42/44 and P42/44 with or without LY294002 from Figure 5c.

Supplementary Figure S8 Examples of Uncropped Western Blots

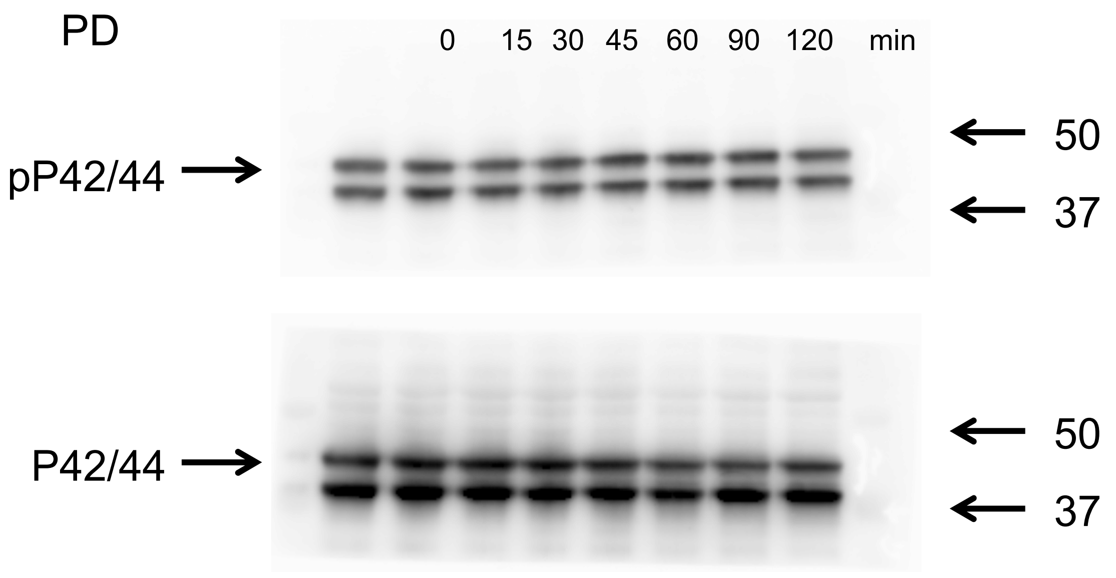

Examples of Uncropped Western Blots for pP42/44 and P42/44 with PD98059 from Figure 5c.

Supplementary Figure S9 Examples of Uncropped Western Blots

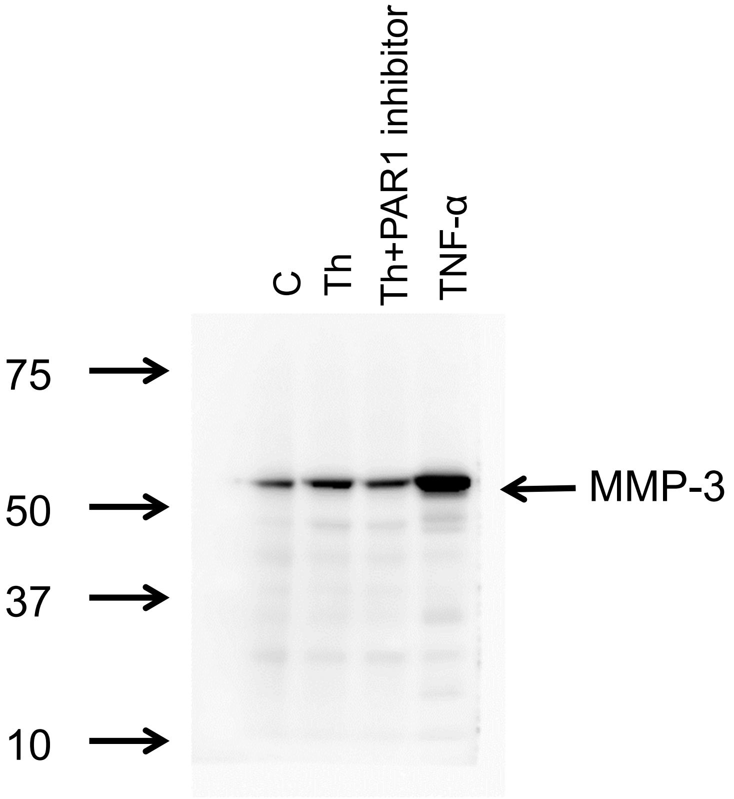

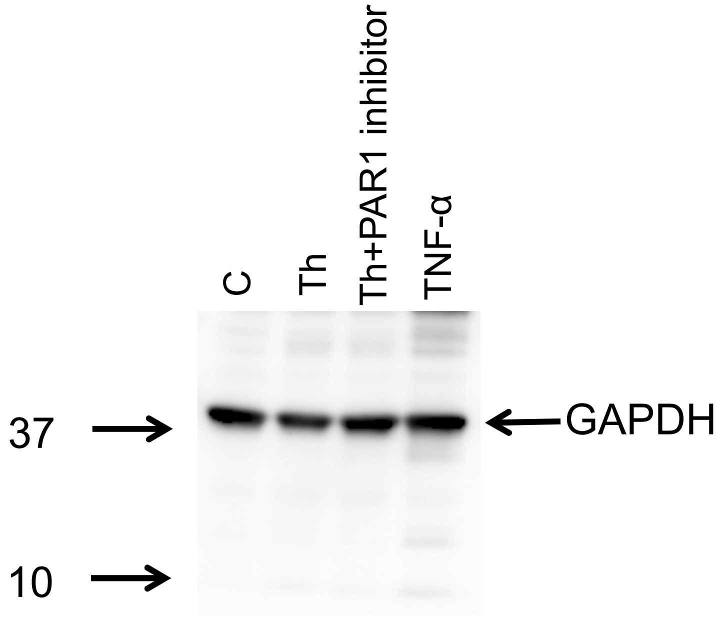

Examples of Uncropped Western Blots for MMP-3 and GAPDH from Figure 6a.
